# Part I of GANNET53: A European Multicenter Phase I/II Trial of the Hsp90 Inhibitor Ganetespib Combined With Weekly Paclitaxel in Women With High-Grade, Platinum-Resistant Epithelial Ovarian Cancer—A Study of the GANNET53 Consortium

**DOI:** 10.3389/fonc.2019.00832

**Published:** 2019-09-10

**Authors:** Isabelle Ray-Coquard, Ioana Braicu, Regina Berger, Sven Mahner, Jalid Sehouli, Eric Pujade-Lauraine, Philippe Alexandre Cassier, Ute Martha Moll, Hanno Ulmer, Karin Leunen, Alain Gustave Zeimet, Christian Marth, Ignace Vergote, Nicole Concin

**Affiliations:** ^1^Centre Anticancereux Léon Bérard, University Claude Bernard Lyon, GINECO Group, Lyon, France; ^2^Department of Gynecology, Charité – Universitätsmedizin Berlin, Corporate Member of Freie Universität Berlin, Humboldt-Universität zu Berlin, Berlin Institute of Health, NOGGO Group, Berlin, Germany; ^3^Department of Obstetrics and Gynecology, Medical University of Innsbruck, Austrian AGO, Innsbruck, Austria; ^4^Department of Gynecology, University Medical Center Hamburg-Eppendorf, AGO, Hamburg, Germany; ^5^Assistance Publique - Hopitaux De Paris, GINECO Group, Paris, France; ^6^Centre Anticancereux Léon Bérard, Lyon, France; ^7^Universitätsmedizin Göttingen, Georg-August-Universität Göttingen, Göttingen, Germany; ^8^Department of Medical Statistics, Informatics and Health Economics, Medical University of Innsbruck, Innsbruck, Austria; ^9^Division of Gynecological Oncology, Department of Gynecology and Obstetrics, Leuven Cancer Institute, Katholieke Universiteit Leuven, Leuven, Belgium

**Keywords:** recurrent ovarian carcinoma, p53 mutation, Hsp90 inhibitors, ganetespib, platinum-resistance

## Abstract

**Background:** Stabilized mutant p53 protein (mutp53) is a novel target in epithelial ovarian cancer. Due to aberrant conformation, mutp53 proteins depend on folding support by the Hsp90 chaperone. Hsp90 blockade induces degradation of mutp53, resulting in tumor cell cytotoxicity and increased sensitivity to chemotherapeutics. Preclinical synergy of the Hsp90 inhibitor ganetespib combined with paclitaxel provided the rationale for testing the combination in platinum-resistant ovarian cancer (PROC) patients in the GANNET53 trial (NCT02012192).

**Methods:** Eligible patients had high-grade PROC with ≤ 4 prior lines of chemotherapy. Weekly paclitaxel (80 mg/m^2^) and increasing doses of ganetespib (100, 150 mg/m^2^) were given i.v. on days 1, 8, 15 in a 28 days cycle until disease progression or unacceptable toxicity. Endpoints were safety and determination of phase II dose. Dose limiting toxicity (DLT) was defined as grade 4 toxicity (with exceptions) occurring in cycles 1&2.

**Results:** Ten patients (median age 59 years; range 43–70) were enrolled. No DLT occurred in cohort 1 (4 patients treated with paclitaxel + ganetespib 100 mg/m^2^), nor in cohorts 2 and 3 (6 patients treated with paclitaxel + ganetespib 150 mg/m^2^). The most common adverse event (AE) related to ganetespib was transient grade 1/2 diarrhea (*n* = 6). Related grade 1/2 AEs in >2 patients included QTc prolongation (*n* = 4), nausea (*n* = 3), anemia (*n* = 3), headache (*n* = 3), fatigue (*n* = 3), and dyspnoea (*n* = 3). Most frequently related grade 3/4 AEs were diarrhea (*n* = 3) and neutropenia (*n* = 2). There was 1 death on study due to hemorrhage from a duodenal ulcer. Three patients discontinued study treatment due to serious AEs (digestive hemorrhage *n* = 1, cardiac failure *n* = 1, abdominal pain and vomiting *n* = 1), 6 due to progressive disease, one due to investigator and patient decision. Two patients achieved a partial response (ORR 20%) and 4 patients a stable disease (disease control rate of 60%). Median PFS was 2.9 months (1.6 months in cohort 1 at 100 mg/m^2^ ganetespib, 5.1 months in cohorts 2+3 at 150 mg/m^2^ ganetespib).

**Conclusions:** The combination of ganetespib 150 mg/m^2^ with paclitaxel 80 mg/m^2^ once weekly for 3 out of 4 weeks was generally well-tolerated with no DLTs, and therefore chosen for the randomized phase II trial.

## Introduction

Epithelial ovarian cancer (EOC) is the most lethal gynecological malignancy. Recent data from the EUROCARE database showed a 5-year relative survival for European women diagnosed with EOC of only 38% (range 31–41% by European region) (1). At the time of diagnosis, most patients have advanced stage disease and despite initial debulking surgery and platinum-based chemotherapy, the majority of patients will relapse and ultimately die of the disease.

A major treatment obstacle in EOC is platinum-resistance. Eventually, most patients will become resistant to platinum after repetitive therapy with platinum-based regimens. Treatment options are limited in patients with platinum-resistant ovarian cancer (PROC). A number of cytotoxic agents including paclitaxel, pegylated liposomal doxorubicin, topotecan, gemcitabine, and cyclophosphamide have shown a relatively modest anti-tumor activity as single agents reflected by low response rates and short-lasting remissions (2–5). Overall, PROC patients face particularly poor outcome with a median PFS of 4 months and a median overall survival (OS) of only 14 months (2–5). There is a pressing need for innovative and more effective treatment strategies to improve survival in ovarian cancer patients with PROC disease.

The vast majority of EOCs are high-grade serous (HGS) carcinomas, which account for 85% of all ovarian cancer deaths (6). Importantly, HGS carcinomas are characterized by the near ubiquitous presence of p53 mutations, their preeminent molecular hallmark (7). The Cancer Genome Atlas Research Network (TCGA) completed whole-exome sequencing on 316 cases of HGS tumors and established that p53 mutations are present in > 96% (8). This strongly suggests that mutp53 is a central oncogenic driver in the pathogenesis of these tumors.

TP53 alterations mainly consist of missense mutations (~74% of all TP53 alterations) (9). Missense mutant p53 proteins (mutp53) accumulate to very high levels in tumor cell nuclei and not only lose their tumor suppressor function, but often acquire new oncogenic functions (gain-of-function, GOF) to actively drive higher proliferation, metastatic ability, and chemoresistance (10–12). Due to their aberrant conformation mutp53 proteins depend on permanent folding support by Hsp90, a cancer-induced multi-component chaperone machinery from the heat shock family (13). This stable interaction between mutp53 and Hsp90 protects mutp53 from degradation by its E3 ubiquitin ligases MDM2 and CHIP and is largely responsible for mutp53 accumulation specifically in tumor cells (14). Pharmacological inhibition of the machine's core ATPase Hsp90 destroys the complex between Hsp90 and mutp53, thereby liberating mutp53 and inducing its degradation by MDM2 and CHIP. As a consequence, in preclinical models Hsp90 blockade shows preferential and strong cytotoxicity for mutp53 cancer cells in culture and in xenografts, as well as in autochthonous mutp53 lymphomas and colon carcinomas in mutp53 knockin mice (15–17). In contrast, wild-type p53 or p53 null cells and tumors show no significant response. Moreover, Hsp90 blockade—by virtue of depleting mutp53—dramatically sensitizes mutp53 cancer cells to chemotherapeutics (18). Given the advanced development of Hsp90 inhibitors, this new paradigm holds immediate strong translational potential in mutp53-driven cancers such as HGS EOC.

We herein report on the first clinical trial applying the highly potent, second-generation Hsp90 inhibitor ganetespib in ovarian cancer patients in general, and in PROC patients in particular. Ganetespib is the clinically most advanced Hsp90 inhibitor which has been applied in more than 1,600 individuals (patients and healthy volunteers) throughout Phase I-III studies. Importantly, ganetespib lacks ocular and liver toxicities which plague first generation ansamycin-type Hsp90 inhibitors and other second-generation Hsp90 inhibitors. The GANNET53 trial applies weekly ganetespib in a new combination with weekly paclitaxel in PROC patients with histological subtypes typically harboring p53 mutations such as HGS, high-grade endometrioid, and undifferentiated carcinoma. This manuscript reports on Part I of the Phase I/randomized Phase II GANNET53 trial. This is a Phase I dose escalation study with a classical 3+3 design aiming to evaluate safety of ganetespib in a new combination with paclitaxel weekly and to determine the ganetespib combination dose to be used in the randomized Phase II trial. The GANNET53 clinical trial is a seventh framework program project (FP7) and fully funded by the European Commission (www.gannet53.eu, grant agreement no 602602).

## Patients and Methods

### Patient Selection

Female patients 18 years or older with epithelial ovarian, fallopian tube, or peritoneal cancer and the histological subtypes high-grade serous, high-grade endometrioid, or undifferentiated carcinoma which typically harbor TP53 mutations were eligible for this trial. All patients had platinum-resistant disease. Both primary platinum-resistant disease (progression > 1 month and ≤ 6 months after platinum-based therapy) or secondary platinum-resistant disease including secondary platinum-refractory disease (progression ≤ 6 months after or during reiterative platinum-based therapy) were allowed. Patients were required to have an Eastern Cooperative Group performance status of 0–1. Adequate bone marrow and organ function was mandatory, defined as absolute neutrophil count ≥ 1.5 × 10^9^/L, platelet count ≥ 100 × 10^9^/L, total bilirubin ≤ 1.5 × upper limit of normal (ULN), AST and ALT ≤ 3 × ULN, and creatinine <2 mg/dl. Patients with disease measurable according to RECIST 1.1 or evaluable by GCIG CA125 criteria were eligible. The main exclusion criteria included primary platinum-refractory disease (progression during primary platinum-based therapy), previous treatments with more than 4 chemotherapy regimens and/or more than 2 chemotherapy regimens in the platinum-resistant setting (excluding targeted and endocrine therapies), peripheral neuropathy ≥ grade 2, clinically active brain metastases, significant cardiac disease (New York Heart Association Class 3 or 4, myocardial infarction within the past 6 months, unstable angina, coronary angioplasty or coronary artery bypass graft within the past 6 months, uncontrolled atrial or ventricular cardiac arrhythmias), a history of prolonged QT syndrome or a family member with prolonged QT syndrome, QTc interval > 470 ms, and ventricular tachycardia or supraventricular tachycardia that require treatment with a Class Ia or Class III antiarrhythmic drug.

This trial was conducted in accordance with the Declaration of Helsinki and the Good Clinical Practice guidelines of the International Conference on Harmonization. Informed consent was obtained from each participant by the investigators, and protocol design and conduct followed all applicable regulations, guidances, and local policies (EudraCT Number: 2013-003868-31; ClinicalTrial.gov Identifier: NCT02012192; DRKS Identifier: DRKS00005501).

### Study Design and Treatment Plan

This is a European multicenter two-part trial (GANNET53), with a part I open-label Phase I dose escalation/de-escalation trial and a traditional 3+3 design, reported here. The phase I study was conducted at five clinical sites in four European countries, i.e., Medical University of Innsbruck, Austria (via the national trial group Austrian AGO; legal Sponsor), Charité Universitätsmedizin Berlin, Germany (via the national trial group NOGGO), Universitätsklinikum Hamburg-Eppendorf, Germany (via the national trial group AGO), Katholieke Universiteit Leuven, Belgium, and Centre Anticancerereux Léon Bérard in Lyon, France (via the national trial group GINECO). A total of 10 PROC patients were included from July 14, 2014 until October 15, 2014. The study was completed on August 26, 2015 (last patient off study). Data lock and closure of the GANNET53 trial (for parts I and II) was performed on December 4, 2017.

Ganetespib was applied once weekly in combination with paclitaxel once weekly for 3 out of 4 weeks (Days 1, 8, 15 of each 4-week cycle) in PROC patients. For evaluation of dose-limiting-toxicity (DLT), each patient was required to receive at least two complete cycles of experimental therapy (DLT observation time-frame), followed by a safety follow-up 28 days (±7 days) after the last administration of the investigational medical product (IMP) ganetespib. Patients were allowed to continue to receive experimental treatment until progression if a benefit for the patient was observed according to the investigators' evaluation.

Paclitaxel weekly was given at a fixed dose level of 80 mg/m^2^ according to the standard weekly scheme in PROC patients_._ The ganetespib dosing scheme and dose levels were chosen based on toxicity data from prior ganetespib single agent studies [Synta-sponsored Phase I studies 9090-01 (*n* = 70) and 9090-02 (*n* = 53)], and ganetespib combination studies with the taxane docetaxel [Synta-sponsored Phase I study 9090-07 (*n* = 27) and Phase IIb/III study 9090-08 (385 patients as of November 13, 2015)]. These studies determined a recommended combination dose for ganetespib of 150 mg/m^2^ once weekly in solid cancers.

A traditional 3+3 design was applied with no intra-patient dose escalation. The ganetespib starting dose in the first cohort of three patients was 100 mg/m^2^. In general, given that one of the first three patients at a given dose level experienced DLT, three more patients were planned to be treated at the same dose level. A single ganetespib dose escalation step was foreseen from 100 to 150 mg/m^2^. Of note, the goal was not to determine the maximum tolerated combination dose, but whether the recommended combination dose of weekly ganetespib of 150 mg/m^2^ in solid tumors is applicable for the new combination with paclitaxel weekly. The dose escalation step was performed in case that DLT occurred in <33% of patients at the starting dose level (corresponding to no patient facing DLT in the first cohort of three, or a maximum of one patient among an extended group of six patients). In case of DLT in <33% of patients at the ganetespib dose level of 150 mg/m^2^, this dose was going to be used in the randomized Phase II trial according to trial protocol. In general, in case of DLT in ≥33% of patients at a given ganetespib dose level, reduction steps to 125, 100, and to 75 mg/m^2^, respectively, were possible according to trial protocol. At the determined dose level to be used in the randomized Phase II trial, extension to a second cohort of another three patients was foreseen.

A Data and Safety Monitoring Committee (DSMC) of four experts in this field was established prior to study start and was responsible to independently evaluate patients' safety throughout the trial. According to trial protocol, at least two meetings of the DSMC were mandatory, one prior to the single dose escalation step of ganetespib and another after completion of the DLT observation time frame in all patients in order to determine the ganetespib dose to be used in the randomized Phase II trial.

### Endpoints, Safety, and Response Assessments

The primary aim of the phase I study was to determine the safety of ganetespib in a new combination with paclitaxel weekly and to determine the ganetespib combination dose to be used in the randomized Phase II GANNET53 trial.

The primary endpoint of the trial was safety assessed by adverse events measured according to NCI CTCAE, version 4.03. For determination of the ganetespib combination dose to be used in the randomized Phase II trial, the endpoint of interest in phase I was whether or not a patient experiences DLT. Table 1 summarizes the applied DLT definition. The observation period for DLT was defined as two complete cycles of treatment, from the first day (D1) of cycle 1 to the last day (D28) of cycle 2.

**Table 1:** Criteria for dose-limiting toxicities (DLTs).

	**Adverse event**	**Grade**	**Comment**	**DLT (yes/no)**
Non-hematologic	Any (with exceptions)	4	Significant, clinically relevant	Yes with the exceptions of alopecia nausea and vomiting without optimal prophylactic measures
	Alopecia	4		No
	Nausea	4		No
	Vomiting	4	Occurred despite optimal prophylactic measures (e.g., antiemesis, loperamide)	Yes
		4	No optimal prophylactic measures applied	No
	Diarrhea	4	Occurred despite optimal prophylactic measures (e.g., loperamide)	Yes
		4	No optimal prophylactic measures applied	No
	Fatigue	4		Yes
		1, 2, 3		No
	Any	3, 4	Not improving to baseline or grade ≤ 1 within 21 days of last treatment dose and despite adequate supportive care/toxicity management	Yes
	Elevation of serum bilirubin	4		Yes
	Elevation of AST, ALT, or ALP		> 10 × ULN	Yes
			> 5–10 × ULN and not improving to ≤ 5 x ULN (grade ≤ 2) by day 7	Yes
			> 5–10 × ULN which improved to ≤ 5 x ULN (grade ≤ 2) by day 7	No
	Any AEs		Related to disease progression or considered to be clearly not study drug-related	No
Hemathologic	Thrombocytopenia	4		Yes
	Thrombocytopenia	3	if not recovered to ≤ 2 by day 7 of AE onset	Yes
		3	if recovered to ≤ 2 by day 7 of AE onset	No
	Neutropenia	4	If lasting ≥7 days	Yes
		4	If lasting <7 days	No
	Febrile neutropenia	Any grade		Yes
Other	Any toxicities		Which are treatment-related and prompt a dose reduction of ganetespib during DLT observation time^*^	Yes
	Laboratory toxicity	3, 4	Considered as clinically insignificant by the principal investigator (PI) or related to an underlying condition	No
	any death		Which is considered possibly related to the study drug (determined by the PI)	Yes
			Which is considered not related to the study drug (determined by the PI)	No
	any AEs		Related to disease progression or considered to be clearly not study drug-related	No
	any dose hold during DLT observation time^*^			No

* *DLT observation time: day 1 of cycle 1 to day 28 of cycle 2*.

Secondary endpoints were objective response rates (ORR) and progression-free survival (PFS). Tumor response or progression was evaluated every 8 weeks (±1 week) for the first 24 weeks and every 12 weeks thereafter until disease progression by CT or MRI scans. Assessments were performed according to RECIST, version 1.1 or by GCIG CA125 criteria in case of non-measurable disease (19, 20). High quality data was achieved by a 100% source data verification by the monitor [Clinical Trial Unit (KKS) of the Medical University of Innsbruck, Austria] in all patients included. Furthermore, quality was assured and clinical sites were supported by the local presence of a monitor at the time of first dosing a patient.

### Statistical Analysis

The 3+3 design is rule-based with a statistical power of > 87% to detect at least one out of three patients with a DLT when the probability for a DLT is 50%. Further analyses in the Phase I study included a description of toxicities by frequency, grade, cycle, and dose. Efficacy measures were reported in per-patient listings. All analyses were done in a descriptive way, but Kaplan-Meier method was applied to describe PFS. Analyses were pre-planned for the safety and per-protocol population.

### Drug Supply for Study and Administration, and Prophylactic Use of Concomitant Medication

Ganetespib and subsequent paclitaxel were administered as separate 1-h infusions. Premedication was given according to hospital standards. Prophylactic medication with loperamide 2 mg was strongly recommended in all patients (given 1–2 h before ganetespib administration, to be repeated every 4 h for the first 12 h). Furthermore, patients were advised to maintain appropriate hydration.

The IMP ganetespib was supplied at no charge by Synta Pharmaceuticals in the Phase I GANNET53 trial. All other costs were covered by the European Commission grant (FP7 project, grant agreement number 602602).

## Results

A total of 10 PROC patients were included in this dose escalation/de-escalation Phase I trial by the Medical University of Innsbruck, Austria (*n* = 1), Katholieke Universiteit Leuven, Belgium (*n* = 4), Universitätsmedizin Berlin Charité, Germany (*n* = 2), Universitätklinikum Hamburg Eppendorf, Germany (*n* = 1), and Centre Anticancereux Léon Bérard, Lyon, France (*n* = 2).

### Patients Characteristics

Patients characteristics are summarized in Table 2.

**Table 2:** Baseline characteristics of patients included into the Phase I GANNET53 trial.

	**Cohort 1 (100 mg/m2) 4 patients included**	**Cohorts 2 + 3 (150 mg/m2) 6 patients included**	**Total of 10 patients included**
Median age (range), years	58 (43–62)	60.5 (52–70)	59 (43–70)
Median time between first diagnosis and enrolment (range), years	2.35 (1.35–5.95)	1.93 (0.9–3.58)	1.93 (0.9–5.95)
ECOG performance status	1 (*n* = 4)	0 (*n* = 5) 1 (*n* = 1)	0 (*n* = 5) 1 (*n* = 5)
Median CA125 at screening (range), U/ml	504.25 (401–2677)	1366.75 (224–4914)	791.25 (224–4914)
Number of previous surgeries for ovarian cancer	0 (*n* = 1) 1 (*n* = 1) 3 (*n* = 2)	1 (*n* = 3) 2 (*n* = 2) 3 (*n* = 1)	0 (*n* = 1) 1 (*n* = 4) 2 (*n* = 2) 3 (*n* = 3)
Residual tumor after the latest surgery prior to enrolment, mm	No surgery (*n* = 1) 0 (*n* = 3)	0 (*n* = 2) 3 (*n* = 1) 5 (*n* = 1) 20 (*n* = 2)	No surgery (*n* = 1) 0 (*n* = 5) 3 (*n* = 1) 5 (*n* = 1) 20 (*n* = 2)
High-grade histology	endometrioid (*n* = 1) serous (*n* = 3)	serous (*n* = 6)	endometrioid (*n* = 1) serous (*n* = 9)
Median time to prior chemotherapy (range), months	1.63 (1.47–1.83)	4.03 (1.13–6.73)	2 (1.13–6.73)
Number of total previous chemotherapy lines	2 (*n* = 1) 3 (*n* = 1) 4 (*n* = 2)	1 (*n* = 2) 2 (*n* = 1) 3 (*n* = 2) 4 (*n* = 1)	1 (*n* = 2) 2 (*n* = 2) 3 (*n* = 3) 4 (*n* = 3)
Number of previous chemotherapy lines in platinum-resistance	0 (*n* = 3) 1 (*n* = 1)	0 (*n* = 3) 1 (*n* = 1) 2 (*n* = 2)	0 (*n* = 6) 1 (*n* = 2) 2 (*n* = 2)
Method of tumor response evaluation	Measurable disease by RECIST (*n* = 3) One fast clinical progression (replaced)	Measurable disease by RECIST (*n* = 4) Assessable by GCIG CA125 criteria (*n* = 2)	Measurable disease by RECIST (*n* = 7) Assessable by GCIG CA125 criteria (*n* = 2)

*n, referrers to the number of patients*.

### Course of the Phase I GANNET53 Trial, DLT, and Recommended Dose for Phase II d

**Figure 1:**
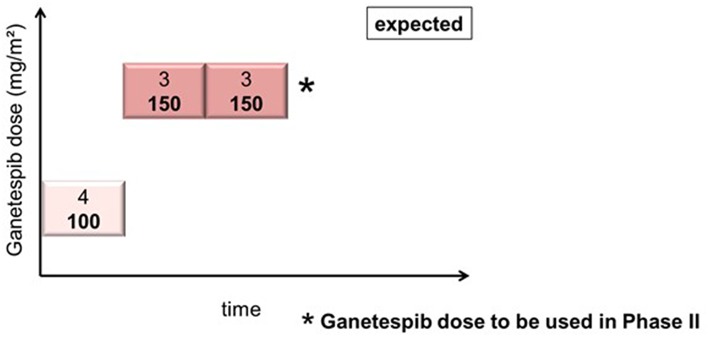
Actual course of the GANNET53 phase I dose escalation/de-escalation trial. Boxes depict patient cohorts and provide information on the number on patients (4 in cohort 1, and 3 in cohorts 2 and 3, respectively) and the dose level of ganetespib (100 mg/m^2^ in cohort 1, and 150 mg/m^2^ in cohorts 2 and 3, respectively). The actual course of the trial took place as expected with one dose escalation step and without necessity for dose de-escalation due to lack of dose-limiting toxicities (DLTs) in the DLT observation time frame of cycles 1 and 2.

In cohort 1 (ganetespib dose level 100 mg/m^2^), one patient had to be replaced based on early disease progression after a single dosing of ganetespib and paclitaxel weekly (cycle 1, day 1). This patient was not evaluable for DLT (DLT observation time-frame minimum of two complete cycles). This resulted in the inclusion of 4 patients in cohort 1. Cohorts 2 and 3 (both at ganetespib dose level 150 mg/m2) consisted of three patients, respectively (Figure 1). No DLT occurred in cohorts 1, 2, and 3.

The DSMC reviewed safety data of patients included into cohort 1 prior to the dose escalation step and concluded that there are no objections to continuing the study according to protocol. After all patients completed the DLT observation time-frame of 2 complete treatment cycles the DSMC concluded that the GANNET53 study can move forward to Phase II without any major concerns. The DSMC recommended to use a weekly ganetespib dose of 150 mg/m^2^ in combination with weekly paclitaxel 80 mg/m^2^ in the randomized Phase II trial.

### Safety

Incidences of grade 1/2 adverse events (AEs) which occurred in more than 1 patient and all ≥ 3 AEs are listed in Table 3.

**Table 3:** Summary of grade 1/2 adverse events (AEs) occurring in > 1 patient and all grade ≥ 3 AEs.

**Grade 1/2 AEs**			**Grade 3/4 AEs**		
**Reported term**	Number of patients affected and relatedness^*^ to ganetespib (*n* = 10)	**Total number of events (*n*)**	**Reported term**	Number of patients affected and relatedness^*^ to ganetespib (*n* = 10)	**Total number of events (*n*)**
Diarrhea	6, related **6**	56	Diarrhea	3, related **3**	5
QT corrected interval prolonged	6, related 4^**^	11	Neutropenia	2, related **2**	2
Nausea	6, related **3**	6	Anemia	3, related **1**	3
Headache^1^	5, related **3**	7	Asthenia	1, related **1**	1
Fatigue	3, related **3**	3	Acute cardiac insufficiency stage IV	1, related **1**	1
Anemia	3, related **3**	3	Gastroduodenal hemorrhage & Hemorrhagic shock from an ulcer duodeni	1, related **1**	1
Dyspnea	3, related **3**	3	Syncope	1, related **1**	1
Anorexia	3, related **2**	3	Pain	1, related 0	1
Peripheral neuropathy	2, related **2**	2	Vomiting	1, related 0	1
Edema peripheral	2, related **2**	2	Polyneuropathy	1, related 0	1
Weight loss	2, related **2**	2	Subileus^4^	2, related 0	2
Abdominal pain^2^	5, related **1**	5	Placement of Tenckhoff catheter	1, related 0	1
Dysgeusia	2, related **1**	2	Ascites	2, related 0	4
Alopecia	2, related **1**	2			
Pain^3^	3, related 0	4			
Asthenia	2, related 0	2			
Pruritus	2, related 0	2			
Subileus^4^	2, related 0	2			
Constipation	2, related 0	5			

1 *Includes: Migraine*.

2 *Includes: Abdominal cramping, Abdominal pain with vomiting*.

3 *Includes: Pain in extremity (lower), Pain leg*.

4 *Includes: Small bowel subobstruction, Obstruction*.

* *Relatedness as evaluated by local PI; AEs categorized as related to study treatment included possibly, probably or definitely related*.

** *For QT prolongation central reviewed of all data were performed by the Sponsor and relatedness as evaluated by Sponsor is given; AEs categorized as related to study treatment included possibly, probably or definitely related*.

The most common AE related to ganetespib was a transient grade 1/2 diarrhea (*n* = 6/10 patients). Furthermore, related grade 1/2 AEs occurring in more than 2 patients were QTc prolongation (*n* = 4), nausea (*n* = 3), anemia (*n* = 3), headache (*n* = 3), fatigue (*n* = 3) and dyspnoea (*n* = 3). Related grade 3/4 AEs were diarrhea (*n* = 3), neutropenia (*n* = 2), anemia, asthenia, syncope and acute cardiac insufficiency (*n* = 1, respectively). There was 1 death on study (after DLT period) caused by digestive tract hemorrhage from a duodenal ulcer. Three patients discontinued study treatment due to serious adverse events (SAEs; digestive hemorrhage *n* = 1, cardiac failure *n* = 1, abdominal pain and vomiting *n* = 1), 6 patients due to progressive disease, and one patient due to physicians' decision.

#### Serious Adverse Reactions

Five SAEs related to ganetespib were reported, i.e., serious adverse reactions (SARs), and are summarized in Table 4.

**Table 4:** Serious adverse reactions (SARs).

**Reported term**	**Grade**	**Ganetespib dose (mg/m^**2**^)**	**Relatedness to ganetespib evaluated by Investigator/Sponsor**	**Outcome**
Gastroduodenal hemorrhage & Hemorrhagic shock from an ulcer duodeni	5 (SUSAR^*^)	150	Unlikely related/ possibly related	Fatal
Acute cardiac insufficiency stage IV^1^	4 (SUSAR^*^)	150	Probably related/ possibly related	Recovered with sequelae
Diarrhea	2	100	Definitely related/ definitely related	Complete recovery
Dyspnea	2	150	Possibly related/ possibly related	Complete recovery
Abdominal pain with vomiting	2	150	Possibly related/ possibly related	Complete recovery

1 *Cardiac insufficiency NYHA stage II-III; loss of systolic LV-function; atrial fibrillation*.

* *Two exclusive SUSARs reported in this Phase I trial*.

One SAR occurred in a 71-year old patient who died from a gastroduodenal hemorrhage and hemorrhagic shock originating from an ulcer in the duodenum. This patient was initially hospitalized for hypotension, hypovolemia, and grade 3 anemia. In the course of hospitalization the situation worsened and hematochezia (with normal colonoscopy findings) and repeated vomiting of blood occurred. The patient received blood transfusions, medication with proton pump inhibitors and repeated emergency gastroscopies were performed. A duodenal bleeding was identified on gastroscopy which was impossible to stop. Ten days after hospitalization the patient died of a hemorrhagic shock. Autopsy confirmed gastrointestinal bleeding from a postpyloric ulcer with a central eroded vessel and an adhesive thrombus on the surface. Microscopic peritoneal carcinosis was present. This event was considered a suspected unexpected serious adverse reaction (SUSAR).

Another SAR occurred in a 61-year old patient who presented with acute cardiac insufficiency stage IV, loss of systolic left ventricular function and atrial fibrillation. This event occurred on day 1 of cycle 3 at the end of the paclitaxel infusion given after the ganetespib infusion. This patient suffered severe underlying conditions such as stage IV chronic renal failure (GFR of 30 ml/min), preceding acute kidney failure 1 year ago, history of renal cell carcinoma (left nephrectomy) and hypertension. Also, the patient received previous angiotensin II receptor antagonist medication and beta-blockers suggesting pre-existing cardiovascular disease. A hydropic heart decompensation due to volume/chemotherapy was suspected by the cardiologists. The Sponsor evaluated this event as confounded by the study medication in addition to the multiple severe underlying conditions. Volume overload during treatment administration and a hypertensive crisis occurring after the paclitaxel infusion might possibly have contributed to the acute heart failure in this patient. In the follow-up this patient has recovered to a left ventricular ejection fraction (LVEF) of 55% (at screening LVEF of 60%). This SAE was assessed as SUSAR.

Three SARs involved grade 2 AEs resulting in hospitalizations, and were therefore judged as serious. This consisted of two cases of one-day hospitalizations, one for grade 2 transient diarrhea, in which the recommended prophylactic loperamide was not given, and one for grade 2 dyspnea occurring 4 days after experimental treatment. Both patients were discharged the next day with complete recovery from symptoms. A third case concerned grade 2 abdominal pain and vomiting, for which the patient was hospitalized in an external hospital, not involved in the conduct of this Phase I study. A laparotomy was performed in which peritoneal carcinomatosis was seen and adhesiolysis and repair of a para-stomal hernia was performed. After 12 days of hospitalization the patient was completely recovered and discharged.

#### Adverse Events of Particular Interest

##### Diarrhea

The most frequent and well-known AE associated with the use of ganetespib is diarrhea which is typically low-grade and transient, lasting 24–48 h after ganetespib administration. Prophylactic medication with loperamide was strongly recommended in all patients. 9/10 patients included in this study experienced at least low-grade diarrhea, which followed the classical transient course. In 3/10 patients grade 3 diarrhea occurred. One of these 3 patients had a pre-existing short bowel syndrome with constant grade 1 diarrhea prior to study inclusion. After each ganetespib application diarrhea worsened transiently, one time to grade 3 diarrhea.

##### QT prolongation

The results of a thorough QT study conducted in healthy volunteers (Study 9090-13) reported a maximum mean ΔΔQTcF of 21.5 ms at 24 h post study drug administration. This finding places ganetespib in a zone of clinical ambiguity. In the present trial echocardiography (ECG) assessments were performed during screening (average of triplicate ECG recording) on day 1 of each treatment cycle and 24-h post-ganetespib-dose on day 2 of cycle 1. Further 24-h post-ganetespib-dose ECGs were strongly recommended to be performed on day 2 of each subsequent cycle. Guidelines were provided in the study protocol for additional intensive ECG monitoring in case of QT prolongation. A thorough review of QT times in all Phase I patients was performed by the Sponsor. Solely grade 1 QT prolongations occurred in the Phase I GANNET53 trial in a total of 6/10 patients. In 4 of these patients the QT prolongation was possibly or probably related to ganetespib. All 4 patients had already pre-existing grade 1 QT prolongation at the time of screening or before their first ganetespib dose, which increased after ganetespib application, yet remained within the grade 1 range. Two patients had pre-existing grade 1 QT prolongation which did not worsen after ganetespib application. In the 4 patients with QT prolongation related to ganetespib a total of 8 events occurred with a median ΔΔQTcF of 21.4 ms (range 8–32 ms) at 24 h post study drug administration.

### Treatment Exposure and Clinical Activity

An overview on treatment exposure and clinical activity is provided in Table 5.

**Table 5:** Treatment exposure and clinical activity.

	**Patient no**	**No. of started cycles**	**Duration of treatment (months)**	**PFS (months)**	**Best overall response (Best OR)**	**Evaluation of the best OR by**	**End of treatment (EOT) reason**
100 mg/m^2^	1	2	1.4	1.6	Progressive disease	RECIST	Progression of disease
	2	2	1.6	1.8	Progressive disease	RECIST	Progression of disease
	3	1	one dose only	0.5	Progressive disease	*immediate clinical progression*	Progression of disease
	4	2	1.6	1.7	Progressive disease	RECIST	Progression of disease
150 mg/m2	5	3	2.3	2.8	Stable disease	GCIC CA125	SAE (SUSAR)
	6	10	9.2	9.3	Stable disease	RECIST	Progression of disease
	7	6	5.3	7.9	Partial response^*^	RECIST	Investigator and patient decision
	8	2	1.6	5.0	Stable disease	RECIST	SAE
	9	3	1.8	4.4	Stable disease	RECIST	SAE (SUSAR)
	10	11	10.1	10.3	Partial response	GCIC CA125	Progression of disease

* *Confirmed response*.

A total number of 42 treatment cycles (median: 2.5 per patient, range 1–11) were applied in the Phase I GANNET53 patients. Of 42 treatment cycles, 35 (83%) cycles were completed with study medication given on all 3 days (D1, D8, D15). The median treatment duration was 1.7 months (range: 1 day−10.1 months). The patient who continued the experimental treatment the longest received 11 cycles of treatment.

ORR was 20% (2/10 patients). Two patients showed a partial remission (one assessed by RECIST, one by CA125 criteria due to non-measurable disease). The two responses lasted 8.5 and 6 months, respectively. Stable disease was seen in 4 patients, resulting in a disease control rate of 60% (6/10 patients). Both partial responses and all stable diseases occurred in the two cohorts with the escalated dose level of 150 mg/m^2^ ganetespib.

**Figure 2:**
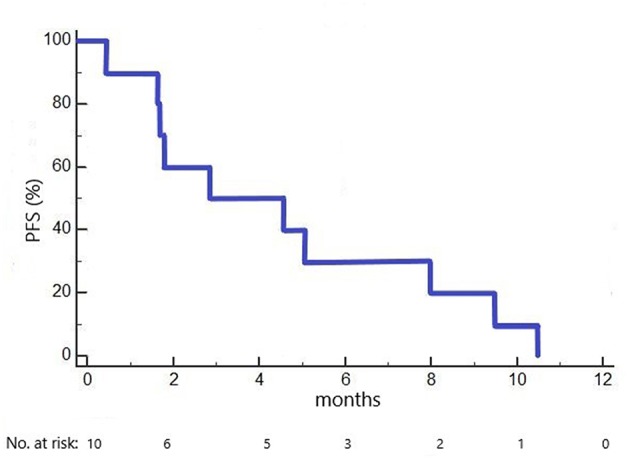
Kaplan-Meier plot of progression-free survival (PFS) in the GANNET53 Phase I trial. All 10 patients included experienced disease progression (no censored cases).

Median PFS in the 10 included patients was 2.9 months (1.6 months in cohort 1 dosed with 100 mg/m^2^ ganetespib, 5.1 months in cohorts 2+3 dosed with 150 mg/m^2^ ganetespib; Figure 2). Three patients had a PFS of > 6 months (7.9, 9.3 and 10.3 months, respectively).

## Discussion

The main objectives of this phase I study were (i) to evaluate the safety of the clinically most advanced Hsp90 inhibitor ganetespib in combination with a standard treatment option in platinum-resistant ovarian cancer patients, namely weekly paclitaxel, and (ii) to determine the ganetespib dose to be applied in combination with paclitaxel in the randomized Phase II GANNET53 trial. Furthermore, first clinical data on preliminary activity were collected. The GANNET53 trial is the first to use ganetespib in ovarian cancer patients, to combine ganetespib with paclitaxel, and to potentially target stabilized mutant gain-of function p53 proteins via the innovative mechanism of induced depletion via Hsp90 inhibition. This study demonstrated that ganetespib 150 mg/m^2^ can be safely combined with paclitaxel 80 mg/m^2^ administered once weekly for 3 out of 4 weeks, and showed first signs of clinical activity in ovarian cancer patients.

Ganetespib is a novel synthetic small molecule and a second-generation Hsp90 inhibitor, which to date has been applied in more than 1,600 individuals within 41 clinical studies. Diarrhea is the most frequent AE associated with ganetespib. The postulated mechanism of action for ganetespib-induced diarrhea is inhibition of EGFR in enterocyte cells that line the gastrointestinal tract, leading to a transient secretory diarrhea, typically limited to 24–48 h following ganetespib infusion. A high rate of diarrhea (90%) occurred in the ovarian cancer patients treated in this study. Diarrhea was typically low-grade and transient. In three patients grade 3 diarrhea occurred which was not classified as serious by the investigator. Particular attention has been paid to this side effect in the present study since the main disease-related symptoms of ovarian cancer patients in general, and in platinum-resistance disease in particular, are also gastrointestinal in nature and related to the uniform presence of peritoneal carcinomatosis (21). Patients in the present study were invited to fill in provided diaries on diarrhea and prophylactic loperamide intake, which was strongly recommended in all patients. High patient compliance was achieved. In a Phase II study in non-small cell lung cancer (NSCLC) 123 patients were treated with a combination of ganetespib and the taxane docetaxel after prophylactic loperamide intake. This study revealed that 45% of NSCLC patients experienced at least one episode of diarrhea of all grades (22). The significantly higher diarrhea rate reported in our study might be related to both, a different patient population and the high attention payed to diarrhea from patients' and investigators' perspectives in our trial. The adverse event diarrhea was manageable with prophylactic and therapeutic loperamide in the treated ovarian cancer population.

Grade 3 neutropenia was seen in 2/10 patients treated in this study, and thus was the second most prevalent ≥ grade 3 AE after diarrhea. No febrile neutropenia occurred. Single agent ganetespib studies showed no evidence of a myelosuppressive effect. In a randomized (1:1) Phase II study in NSCLC comparing ganetespib with docetaxel to docetaxel alone neutropenia was the most frequent grade 3/4 event occurring in 41% of patients in the combination arm, and in 42% in the docetaxel arm, respectively (22). Of note, 10 patients in the combination arm experienced febrile neutropenia vs. 4 patients in the control arm. Though our data in ovarian cancer patients and the combination data of ganetespib and docetaxel in NSCLC do not show a significant increase in the frequency or severity of neutropenia over what would be expected with taxane alone, febrile neutropenia remains an important potential risk.

Another AE of specific interest was QT prolongation with intensified ECG monitoring implemented in the part I GANNET53 trial. None of the 4 patients who experienced a QT prolongation related to ganetespib showed a *de-novo* QT prolongation. All of them had a pre-existing QT prolongation prior to the first ganetespib application. Measured ΔΔQTcF values in 24 h post dose ECGs were in line with results of a thorough QT study conducted in healthy volunteers (Study 9090–13) and all reported events were exclusively grade 1. It is not clear that this finding confers a substantially increased risk of torsades de pointes type or ventricular tachycardia in patients being treated with ganetespib. Importantly, to date no patient treated with ganetespib had torsade de point nor severe ventricular arrhythmias reported on any ECG.

Of note, no ocular or liver toxicity was reported in the 10 ovarian cancer patients treated with this new drug combination. Ocular toxicity, manifested as visual disturbances, has been reported with high prevalence in the range of 50–89% for several other Hsp90 inhibitors and significantly hampered their clinical application (23–25). Visual disturbances in patients treated with the Hsp90 inhibitors 17-DMAG or AUY922 have been linked to the induction of apoptosis in cells in the outer nuclear layer of the retina (26). In contrast, ganetespib did not elicit induction of apoptosis in preclinical studies, which is consistent with the very low number of reported visual disturbance cases in patients. In NSCLC patients treated with ganetespib in combination with docetaxel, visual impairment was experienced in <1% of patients (22). Another potential class effect of Hsp90 inhibitors is elevation in liver enzymes (27). Liver toxicity in the 1st generation geldanamycin-derivatived Hsp90 inhibitors is an off-target effect. The presence of a benzoquinone moiety in those molecules is suspected to cause the liver toxicity, which ganetespib does not contain. Therefore, liver toxicity is not expected in ganetespib. This correlates with the data collected here for the combination with paclitaxel and the general safety information collected to date.

Supported by preclinical data showing synergistic anti-proliferative effects combining ganetespib with paclitaxel in cultured cancer cells and in cancer xenografts *in vivo*, we applied this combination in ovarian cancer patients (28). Paclitaxel given as single agent on a weekly basis at a dose of 80–90 mg/m^2^ proved to be one of the most effective regimens in PROC patient with response rates in the range of 20–60% (2, 3, 29). The AURELIA trial reported significant benefit in terms of PFS using a combination of chemotherapy with bevacizumab for PROC patients who had not received bevacizumab before (3). However, the majority of patients now receive bevacizumab before they develop platinum-resistance. In our Phase I study here clinical activity was noted in cohorts 2+3 receiving 150 mg/m2 ganetespib in combination with paclitaxel, and the activity was in the range of what can be expected with current standard treatment options in PROC patients.

The strength of this Phase I trial are safety and clinical activity data on ganetespib and the combination of ganetesbip with paclitaxel in ovarian cancer, and its high data quality. Furthermore, this is the first clinical trial potentially targeting stabilized mutant gain-of function p53 protein via the mechanism of depletion by HSP90 inhibition. Its limitations are the small number of patients and the lack of integrated pharmacokinetic and pharmacodynamic analyses. Both were included into part II of the GANNET53 trial which has the advantage of a fixed ganetespib dose level and a higher number of patients.

In summary, this study identified ganetespib 150 mg/m^2^ and paclitaxel 80 mg/m^2^ administered once weekly for 3 out of 4 weeks as the recommended phase II dose for PROC patients. The toxicity profile was consistent with the safety profile of each individual agent and was manageable.

## Data Availability

The raw data supporting the conclusions of this manuscript will be made available by the authors, without undue reservation, to any qualified researcher.

## Ethics Statement

This trial was conducted in accordance with the Declaration of Helsinki and the Good Clinical Practice guidelines of the International Conference on Harmonization. Informed consent was obtained from each participant by the investigators, and protocol design and conduct followed all applicable regulations, guidances, and local policies. The protocol was approved by the (1) Ethikkommission der Medizinischen Universität Innsbruck, Austria (AN2013-0071), (2) Ethikkommission der Ärztekammer Hamburg, Germany (PVN4676), (3) Comite de Protection des Personnes- Ile de France 1, Paris, France (2014-fevrier-13492), and (4) Commissie Medische Ethiek van de Universtitaire Ziekenhuizen KULeuven, Belgium (ML10218).

## Author Contributions

IR-C, IB, SM, JS, EP-L, PC, KL, AZ, CM, IV, and NC designed this clinical trial and enrolled patients. HU was the trial statistician and substantially involved in trial planning and analysis. RB administratively coordinated the trial in all participating countries in her function as administrative head of the coordinating national trial group. NC was de coordinating investigator and also coordinator of the FP7 project GANNET53. UM has generated the robust research findings which built the basis and scientific rational for this clinical trial and was involved in all steps of clinical trial planning. IR-C and NC wrote the manuscript. All authors have revised the manuscript and approved it for publication.

### Conflict of Interest Statement

The authors declare that the research was conducted in the absence of any commercial or financial relationships that could be construed as a potential conflict of interest.
